# Supplementary material for: Resolving the full spectrum of human genome variation using Linked-Reads
Source: Genome Res. 2019 Apr;29(4):635–45. doi: 10.1101/gr.234443.118 (PMC6442396; doi:10.1101/gr.234443.118)
Supplement: Supplemental Material [file supp_gr.234443.118_Supplemental_longranger-2.2.zip › longranger-2.2.tar.bz2!/lr2.2-public/lib/stan/cmdstan/stan/doc/api/html/agrad_2fwd_2functions_2fdim_8hpp_source.html]

Stan: src/stan/agrad/fwd/functions/fdim.hpp Source File


|  |  |
| --- | --- |
|  | Stan  2.5.0  probability, sampling & optimization |


- Main Page
- Related Pages
- Namespaces
- Classes
- Files

- File List
- File Members

All Classes Namespaces Files Functions Variables Typedefs Enumerations Enumerator Friends Macros Pages

- src
- stan
- agrad
- fwd
- functions

fdim.hpp

Go to the documentation of this file.

1 #ifndef STAN\_\_AGRAD\_\_FWD\_\_FUNCTIONS\_\_FDIM\_HPP

2 #define STAN\_\_AGRAD\_\_FWD\_\_FUNCTIONS\_\_FDIM\_HPP

3

4 #include <stan/agrad/fwd/fvar.hpp>

5 #include <stan/meta/traits.hpp>

6 #include <stan/math/functions/fdim.hpp>

7

8 namespace stan {

9

10  namespace agrad {

11

12  template <typename T>

13  inline

14  fvar<T>

15  fdim(const fvar<T>& x1, const fvar<T>& x2) {

16  using stan::math::fdim;

17  using std::floor;

18  if(x1.val\_ < x2.val\_)

19  return fvar<T>(fdim(x1.val\_, x2.val\_), 0);

20  else

21  return fvar<T>(fdim(x1.val\_, x2.val\_),

22  x1.d\_ - x2.d\_ \* floor(x1.val\_ / x2.val\_));

23  }

24

25  template <typename T>

26  inline

27  fvar<T>

28  fdim(const fvar<T>& x1, const double x2) {

29  using stan::math::fdim;

30  using std::floor;

31  if(x1.val\_ < x2)

32  return fvar<T>(fdim(x1.val\_, x2), 0);

33  else

34  return fvar<T>(fdim(x1.val\_, x2), x1.d\_);

35  }

36

37  template <typename T>

38  inline

39  fvar<T>

40  fdim(const double x1, const fvar<T>& x2) {

41  using stan::math::fdim;

42  using std::floor;

43  if(x1 < x2.val\_)

44  return fvar<T>(fdim(x1, x2.val\_), 0);

45  else

46  return fvar<T>(fdim(x1, x2.val\_), x2.d\_ \* -floor(x1 / x2.val\_));

47  }

48  }

49 }

50 #endif

stan::agrad::fvar

**Definition:** fvar.hpp:13

stan::agrad::floor

fvar< T > floor(const fvar< T > &x)

**Definition:** floor.hpp:15

stan::math::fdim

boost::math::tools::promote\_args< T1, T2 >::type fdim(T1 a, T2 b)

The positive difference function (C99).

**Definition:** fdim.hpp:23

traits.hpp

fdim.hpp

fvar.hpp

stan::agrad::fvar::d\_

T d\_

**Definition:** fvar.hpp:16

stan::agrad::fdim

fvar< T > fdim(const fvar< T > &x1, const fvar< T > &x2)

**Definition:** fdim.hpp:15

stan::agrad::fvar::val\_

T val\_

**Definition:** fvar.hpp:15


---

[ Stan Home Page ]

*© 2011–2014,
Stan Development Team.*
